# The Effects of Minimal Stimulation Protocol on Preimplantation Genetic Screening

**DOI:** 10.3390/jcm14124285

**Published:** 2025-06-16

**Authors:** Gokalp Oner, Enes Karaman, Ferhan Elmali, Suat Altmisyedioglu, Hande Nur Doganay

**Affiliations:** 1 Department of Obstetrics and Gynecology, Faculty of Medicine, Istanbul Aydin University, 34295 Istanbul, Turkey; 2 IVF Center, Kayseri System Hospital, 38070 Kayseri, Turkey; 3 Department of Obstetrics and Gynecology, Faculty of Medicine, Nigde Omer Halisdemir University, 51120 Nigde, Turkey; 4 Department of Biostatistics, Faculty of Medicine, Izmir Katip Celebi University, 35640 Izmir, Turkey; 5 Obsterics annd Gynecology Clinic, Nigde Omer Halisdemir University Training and Research Hospital, 51100 Nigde, Turkey

**Keywords:** preimplantation genetic screening, advanced maternal age, minimal stimulation protocol, euploidy, embryo transfer

## Abstract

**Background/Objectives:** Preimplantation genetic screening improves embryo selection in intracytoplasmic sperm injection cycles, especially for women of advanced maternal age. As chromosomal normality declines with age, high-dose gonadotropins are commonly used to enhance follicular response. This study compares minimal and high-dose stimulation protocols in terms of euploidy, pregnancy, and live birth rates following single embryo transfer. **Methods**: In this prospective study, 198 women aged 38–45 years were enrolled and divided into two groups: minimal stimulation (100 mg clomiphene citrate and 75 IU human menopausal gonadotropin) and high stimulation (300–450 IU gonadotropins). Women with severe male factor infertility, endometriosis, or absolute tubal factor were excluded. Clinical outcomes were compared using a *t*-test or Mann–Whitney U test. **Results**: Baseline characteristics were similar between groups. The high-dose group had a significantly higher number of retrieved oocytes (*p* = 0.009) and metaphase II oocytes (*p* = 0.003). However, there were no significant differences in euploid embryo rates (35.4% vs. 37.4%, *p* = 0.768), clinical pregnancy rates (67.6% vs. 69.4%, *p* > 0.999), gestational sac rates (58.8% vs. 58.3%, *p* > 0.999), or live birth rates (47.1% vs. 50.0%, *p* = 0.995). **Conclusions**: This is the first prospective study to compare euploid embryo rates, pregnancy rates, and live birth rates between minimal stimulation protocol and high stimulation protocol in AMA patients. Although there has been no difference in euploid and pregnancy rates, minimal stimulation protocol has advantages in cost and comfort.

## 1. Introduction

Infertility, defined as the inability to conceive after 12 months of unprotected intercourse, is a growing health concern affecting both individuals and societies worldwide [1]. It can arise from a multitude of factors, including ovulatory dysfunction, diminished ovarian reserve, male factor infertility, tubal pathology, and unexplained causes. In women, one of the most critical determinants of reproductive potential is age, as both the quantity and quality of oocytes decline with advancing years [2]. Advancing age not only reduces the probability of natural conception but also increases the risk of chromosomal abnormalities and pregnancy complications [3]. As such, understanding and optimizing fertility treatments in aging populations has become a priority in reproductive medicine.

Globally, infertility affects an estimated 15% of couples, translating to approximately 48 to 186 million individuals [4]. Among women, the impact of age-related infertility is particularly pronounced. According to the Centers for Disease Control and Prevention (CDC), nearly one in five women in the United States experience difficulties getting pregnant after the age of 35 [5]. In high-income countries, the median age of first-time mothers has steadily increased, with many seeking assisted reproductive technologies (ART) in their late 30s or early 40s [6]. Despite technological advancements, the success rates of ART decrease significantly with age. For example, live birth rates per cycle drop from around 40% in women under 35 to less than 10% in women over 42 [7]. These statistics underscore the importance of developing effective, age-appropriate fertility treatments that address the unique challenges of women of advanced maternal age (AMA).

In this context, preimplantation genetic screening (PGS) has emerged as a pivotal tool for selecting chromosomally normal embryos, particularly in intracytoplasmic sperm injection (ICSI) cycles, where it is essential to identify euploid embryos that are more likely to result in viable pregnancies [8]. Traditionally, high-dose gonadotropin stimulation protocols have been the standard approach to enhance oocyte retrieval in women of AMA, with the primary goal of maximizing the number of eggs available for fertilization and genetic testing. These protocols are designed to induce a robust ovarian response, increasing the chances of obtaining a viable, euploid embryo. However, despite the potential benefits of maximizing oocyte yield, these high-dose protocols are associated with several challenges, including an increased risk of ovarian hyperstimulation syndrome (OHSS), higher costs, and greater patient discomfort due to the invasive nature of the treatment [9].

Furthermore, research has demonstrated that the number of oocytes retrieved does not always correlate with improved embryo quality or clinical outcomes, particularly in women of advanced maternal age [10]. While a higher oocyte yield may provide more embryos for genetic screening, it does not necessarily improve the rate of euploid embryos, as the age-related decline in oocyte quality remains a major limiting factor [11]. Consequently, there is growing interest in exploring alternative, less invasive protocols that may offer similar or even superior reproductive outcomes with fewer associated risks.

One promising alternative to high-dose stimulation is the minimal stimulation protocol (MSP), which uses lower doses of gonadotropins, often in combination with oral agents such as clomiphene citrate (CC) or letrozole. MSPs are designed to produce fewer but potentially higher-quality oocytes, with the goal of preserving the natural hormonal environment while reducing patient burden. Unlike high-dose protocols, MSPs aim to minimize the use of exogenous gonadotropins, thereby decreasing the risk of OHSS and reducing the financial and emotional cost of treatment [12,13]. The rationale behind MSP is based on the hypothesis that ovarian stimulation at physiological or near-physiological levels may be more conducive to producing euploid embryos in older women by reducing the potential negative effects of supraphysiologic hormone levels on oocyte quality and endometrial receptivity [14].

Recent studies have begun to explore the feasibility of MSPs in women of AMA, with promising results suggesting that MSPs may offer comparable embryo euploidy rates, clinical pregnancy rates, and live birth outcomes to those achieved with high-dose protocols [15,16]. For example, several studies have demonstrated that while MSPs typically yield fewer oocytes, they do not appear to compromise clinical success, with some studies even showing higher embryo quality and better implantation rates compared to high-dose stimulation protocols [17,18]. In contrast, high-dose protocols have often been associated with higher numbers of oocytes retrieved but no corresponding improvement in embryo quality or clinical pregnancy outcomes [19,20].

However, despite these promising findings, the majority of studies comparing MSPs to high-dose protocols have been retrospective or observational in nature, leaving a gap in the literature regarding the direct comparison of these two approaches in a prospective setting. This gap is particularly notable in women of AMA, who represent a unique challenge in ART due to their diminished ovarian reserve and increased risk of aneuploidy. In light of this, prospective studies are essential to provide more robust evidence on the comparative efficacy of MSPs and high-dose stimulation protocols in this population. Furthermore, while previous studies have focused on the clinical and embryological outcomes of different stimulation protocols, few have considered the patient-centered aspects of treatment, such as the impact of stimulation protocols on patient well-being, cost-effectiveness, and overall satisfaction.

Considering these research gaps, the current study aimed to evaluate the impact of a minimal stimulation protocol using CC and hMG on euploidy rates, clinical pregnancy rates, and live birth outcomes in comparison with traditional high-dose gonadotropin stimulation in AMA women. By addressing both clinical efficacy and patient comfort, this research contributes to the ongoing discussion regarding optimal ovarian stimulation strategies in ART.

## 2. Materials and Methods

### 2.1. Study Design and Participants

This prospective cohort study was conducted as a dual-center investigation, primarily at the Kayseri System Hospital IVF Center. The study included a total of 198 women of advanced maternal age (AMA), between 38 and 45 years old, undergoing ICSI cycles with preimplantation genetic screening (PGS). While the primary data source was the Kayseri System Hospital IVF Center, additional clinical data were also obtained from Nigde Omer Halisdemir University Training and Research Hospital, which actively contributed to both patient recruitment and clinical follow-up as part of the study protocol.

The study was approved by the Ethics Committee of Nigde Omer Halisdemir University (Ethics No: 2025/07-88) and registered on ClinicalTrials.gov (ID: NCT05220995). Written informed consent was obtained from all participants prior to enrollment.

Inclusion criteria comprised women aged 38–45 years with normal uterine anatomy and undergoing controlled ovarian stimulation for PGS. All patients in the clinic were followed by G.O., and all met the Bologna criteria for poor ovarian response. The Bologna criteria, established by the European Society of Human Reproduction and Embryology (ESHRE), define poor ovarian responders as patients who meet at least two of the following three features:

1. Advanced maternal age (≥40 years) or other risk factors for POR.
2. A previous poor ovarian response (≤3 oocytes retrieved with conventional stimulation).
3. Abnormal ovarian reserve tests, such as the following:
  - Antral follicle count (AFC) < 5–7;
  - Anti-Müllerian hormone (AMH) < 0.5–1.1 ng/mL.

Patients must meet at least two of these three criteria to be classified as poor responders [21].

This cycle of all treatments is the first attempt in our center.

Exclusion criteria included severe male factor infertility, diagnosed endometriosis, and absolute tubal factor infertility.

### 2.2. Ovarian Stimulation Protocols

Participants were stratified into two groups based on the ovarian stimulation protocol used:

MSP group: Women received clomiphene citrate (100 mg/day) starting on day 3 of the menstrual cycle, continued for 5 days, in combination with hMG at a dose of 75 IU/day until the trigger day.

High-dose stimulation protocol (HSP) group: Women received recombinant gonadotropins at an initial dose of 300 IU/day, with a maximum of 450 IU/day, beginning on day 2 or 3 of the cycle. Dose adjustments were made based on follicular response as assessed by transvaginal ultrasound and serum estradiol levels.

Ovulation was triggered when at least two follicles ≥ 18 mm were observed, using recombinant hCG or a GnRH agonist. Oocyte retrieval was performed 36 h post-trigger.

There have been no drug-related side effects in the study groups.

### 2.3. Laboratory Procedures and Genetic Screening

Retrieved oocytes were inseminated via ICSI, and resulting embryos were cultured to the blastocyst stage (Day 5). All of the embryos are undergone biopsies. Embryo biopsies were taken from trophectoderm cells, followed by vitrification of the biopsied blastocysts. Preimplantation genetic screening was performed using next-generation sequencing (NGS) to assess chromosomal euploidy.

### 2.4. Frozen Embryo Transfer (FET)

Only euploid embryos were considered for single embryo transfer in subsequent frozen-thawed cycles. Endometrial preparation was performed with estrogen and progesterone, and endometrial thickness was recorded on the day progesterone was initiated.

### 2.5. Outcome Measures

The primary outcomes included embryo euploidy rate, clinical pregnancy rate (defined as the presence of a gestational sac with fetal heartbeat), and live birth rate. Secondary outcomes included the number of oocytes retrieved, the number of metaphase II oocytes, and endometrial thickness. M2 oocytes are mature oocytes that have reached metaphase II and are suitable for intracytoplasmic sperm injection.

### 2.6. Statistical Analysis

Data were evaluated using the IBM SPSS Statistics Standard Concurrent User V 29 (IBM Corp., Armonk, New York, NY, USA) statistical package program. Descriptive statistics, including the number of units (n), percentage (%), and mean ± standard deviation., were given as median and interquartile distance values. The normal distribution of data belonging to numerical variables was assessed using the Shapiro–Wilk normality test. The variance homogeneity of the groups was analyzed using the Levene test. For numerical variables, two-group comparisons were performed using the independent samples *t*-test if the data showed a normal distribution, and the Mann–Whitney U test was used if it did not show a normal distribution. Pearson chi-square and Yates chi-square tests were used to compare groups with categorical variables. A *p* value of <0.05 was considered statistically significant.

## 3. Results

### 3.1. Baseline Demographic and Hormonal Characteristics

A total of 198 women diagnosed with AMA undergoing PGS cycles were included in the study, with 99 participants in each group. The MSP group received the minimal stimulation protocol, while the HSP group underwent high-dose stimulation. There were no statistically significant differences in the baseline characteristics of the two groups. The mean age was 40.4 ± 1.5 years in the MSP group and 40.2 ± 1.5 years in the HSP group (*p* = 0.383), and body mass index (BMI) was also similar between the groups, with a mean of 25.96 ± 4.93 in the MSP group and 26.45 ± 4.27 in the HSP group (*p* = 0.461).

Hormonal and ovarian reserve markers were also comparable. Anti-Müllerian hormone (AMH) levels had a median of 0.50 ng/mL (interquartile range [IQR]: 0.60) in the MSP group and 0.70 ng/mL (IQR: 0.60) in the HSP group (*p* = 0.278). Baseline day 2 hormone levels were not significantly different, including follicle-stimulating hormone (FSH) (9.26 ± 2.14 vs. 8.74 ± 2.23 mIU/mL; *p* = 0.092), luteinizing hormone (LH) (7.73 ± 1.55 vs. 8.08 ± 1.42 mIU/mL; *p* = 0.096), and estradiol (E2) levels (median 40.00 vs. 33.00 pg/mL; *p* = 0.067). Endometrial thickness on the day of progesterone administration was also statistically similar, measuring 10.66 ± 1.60 mm in the MSP group and 11.11 ± 1.92 mm in the HSP group (*p* = 0.072). The MSP group has statistically significant low dose usage when compared to the HSP group (377.9 ± 135.7 vs. 2817.2 ± 945.0; *p* = 0.001).

However, significant differences were observed in ovarian response between the two stimulation protocols. Antral follicle count (AFC) was significantly higher in the HSP group, with a median of 4.0 follicles (IQR: 2.0), compared to 3.0 follicles (IQR: 2.0) in the MSP group (*p* = 0.013). Similarly, the number of metaphase II (M2) oocytes retrieved was significantly greater in the HSP group, with a median of 4.0 (IQR: 1.0) compared to 3.0 (IQR: 2.0) in the MSP group (*p* = 0.003) (Table 1).

The live birth rate per euploid embryo was 45.7% in the MSP group, and the live birth rate per euploid embryo was 48.6% in the HSP group. There no statistically significant difference was found (*p* = 0.22).

### 3.2. Preimplantation Genetic Screening Outcomes

The analysis of preimplantation genetic diagnosis (PGD) outcomes revealed no statistically significant difference in the rate of euploid embryos between the two groups. In the MSP group, 35 out of 99 embryos (35.4%) were found to be genetically normal, while 64 embryos (64.6%) were aneuploid. In the HSP group, 37 out of 99 embryos (37.4%) were euploid, and 62 (62.6%) were aneuploid (*p* = 0.768). These findings suggest that despite differences in stimulation intensity and oocyte yield, the rate of chromosomally normal embryos remained similar across both protocols.

### 3.3. Clinical and Live Birth Outcomes

Clinical outcomes following embryo transfer also showed no significant differences between the two stimulation protocols. In the MSP group, 23 of the 34 women who underwent embryo transfer (67.6%) achieved a positive beta-hCG test, compared to 25 of 36 women (69.4%) in the HSP group (*p* > 0.999). The presence of a gestational sac, confirming clinical pregnancy on ultrasound, was documented in 20 women (58.8%) in the MSP group and 21 women (58.3%) in the HSP group (*p* > 0.999).

Live birth outcomes were likewise comparable. Sixteen women (47.1%) in the MSP group had live births, compared to 18 women (50.0%) in the HSP group (*p* = 0.995). These results indicate that minimal stimulation protocol achieves similar clinical pregnancy and live birth outcomes when compared to high-dose stimulation, despite the latter yielding a higher number of mature oocytes.

## 4. Discussion

In recent years, fertility treatment strategies have increasingly shifted toward individualized approaches that consider patient characteristics such as age, ovarian reserve, and treatment burden [22]. Among women of advanced maternal age, diminished ovarian reserve and compromised oocyte quality present significant challenges in assisted reproductive technologies [2]. While traditional protocols have relied on high-dose gonadotropins to maximize oocyte yield, emerging evidence suggests that such aggressive stimulation may not necessarily improve outcomes [9].

In the present study, although high-dose stimulation protocols were associated with significantly greater antral follicle counts and metaphase II (M2) oocyte yields, both stimulation strategies achieved statistically comparable outcomes in terms of embryo euploidy, clinical pregnancy rates, and live birth rates. These findings challenge the prevailing clinical assumption that maximizing ovarian stimulation through higher gonadotropin dosages consistently translates into superior reproductive outcomes, particularly in the context of older reproductive-aged women. The results show that despite the higher number of retrieved oocytes in the high-dose group, the rate of euploid embryos was similar between both groups (35.4% in the minimal stimulation group vs. 37.4% in the high-dose group; *p* = 0.768). This supports the hypothesis that the primary determinant of embryo aneuploidy is intrinsic oocyte quality and maternal age rather than stimulation intensity [10].

Similar findings have been reported by Abbara et al. and Ozgur et al., who demonstrated that although high-dose gonadotropin regimens lead to a higher yield of retrieved oocytes and M2 oocytes, they did not improve euploidy rates or cumulative live birth rates [19,20]. These studies suggested that increasing the number of oocytes retrieved may not overcome the inherent chromosomal challenges posed by aging oocytes. Furthermore, Cohen et al. and Cesarano et al. observed that an increased ovarian response does not necessarily correlate with improved clinical outcomes in older women. Authors argued that while high gonadotropin dosages can result in more oocytes, they do not translate into a meaningful increase in the number of euploid embryos and, consequently, do not lead to improved pregnancy rates or live birth outcomes [23,24]. The results from the current study corroborate these findings, as clinical pregnancy rates (67.6% in the minimal stimulation group vs. 69.4% in the high-dose group; *p* > 0.999) and live birth rates (47.1% in the minimal stimulation group vs. 50.0% in the high-dose group; *p* = 0.995) were statistically similar, despite the significant difference in oocyte yield between the groups.

This study also highlights the role of embryo competence and endometrial receptivity in achieving successful implantation and live birth outcomes. The findings suggest that the number of oocytes retrieved does not necessarily dictate clinical success. Indeed, the similar endometrial thickness observed between the two groups indicates that minimal stimulation does not impair endometrial preparation or implantation potential. This is consistent with previous reports suggesting that the success of an IVF cycle is not solely dependent on the number of oocytes but rather on factors such as embryo quality and the receptivity of the endometrium [25]. High gonadotropin regimens may result in supraphysiological hormone levels that could compromise endometrial receptivity, a key factor in implantation success [24,26]. This study suggests that minimal stimulation protocols may better preserve the natural hormonal environment, potentially improving endometrial receptivity without compromising the outcome.

Moreover, study findings support the notion that MSP offers a more patient-friendly approach, with lower costs, reduced physical strain, and fewer potential complications such as ovarian hyperstimulation syndrome, which is associated with high-dose gonadotropin regimens [27]. The lower gonadotropin doses in the MSP may be particularly advantageous for women with poor ovarian reserve or those who are particularly sensitive to hormonal fluctuations [28,29]. In our study, a statistically significant lower gonadotropin dosage was used in the MSP group. According to the literature [30], this may show that MSP is a cost-effective treatment option.

Interestingly, while the results of this study show no significant difference in clinical pregnancy or live birth rates between the two protocols, they contribute to the growing body of evidence that supports minimal stimulation as a viable alternative to conventional high-dose protocols. These findings align with previous studies, which demonstrated that minimal stimulation in older women can yield comparable clinical outcomes with reduced adverse effects [12,13]. As such, the current study further establishes minimal stimulation protocols as a reasonable first-line option for AMA women seeking to preserve their reproductive health and improve their chances of a successful pregnancy outcome.

### 4.1. Study Implications

The results of this study offer significant clinical implications for managing AMA patients undergoing IVF with PGS. Traditionally, high-dose ovarian stimulation has been favored in AMA patients to maximize oocyte yield; however, the study findings suggest that MSP can provide comparable outcomes in terms of embryo euploidy, clinical pregnancy, and live birth rates. This challenges the prevailing notion that more aggressive stimulation necessarily leads to better outcomes. The use of MSP not only reduces the physical and emotional burden on patients but also offers a cost-effective alternative without compromising success. Particularly for AMA patients who may undergo multiple IVF cycles, MSP represents a more sustainable and patient-centered approach. These findings support a broader move toward individualized treatment strategies that prioritize patient comfort and clinical efficiency.

### 4.2. Study Strengths and Limitations

One of the key strengths of this study is its prospective design, which minimizes recall and selection biases commonly associated with retrospective analyses and allows for more accurate and reliable data collection. Additionally, by focusing exclusively on women of advanced maternal age undergoing preimplantation genetic screening with single embryo transfer, the study provides targeted insights into a high-risk population that is often underrepresented in randomized controlled trials. The inclusion of embryo euploidy rates as a primary outcome further strengthens the study, as it offers a direct assessment of chromosomal integrity, a crucial determinant of successful implantation and live birth. Moreover, the standardized stimulation protocols and uniform embryological assessment protocols across both groups enhance the internal validity of the comparisons. Finally, the comprehensive evaluation of both clinical outcomes and laboratory parameters allows for a more nuanced understanding of the relationship between stimulation intensity, embryo competence, and reproductive success in this specific patient population.

Despite the strengths of this prospective study, including its focused cohort and standardized embryo assessment through PGS, several limitations must be acknowledged. Being a single-center study, the results may not be generalizable to all clinical settings or patient populations. The sample size, while sufficient to detect differences in primary outcomes, may have been underpowered to detect subtle variations in secondary outcomes or allow for comprehensive subgroup analyses. Additionally, the study did not include long-term follow-up of offspring, limiting the understanding of the broader impact of different stimulation protocols on child health. By excluding patients with severe male factor infertility, endometriosis, and tubal disease, the study population was somewhat selective, potentially limiting its applicability to more complex infertility cases. Moreover, although only single euploid embryo transfers were included to standardize outcomes, this may not reflect routine practices in all fertility centers, where multiple embryo transfers or different embryo selection strategies might be employed.

### 4.3. Future Recommendations

Future research should aim to validate and expand upon these findings through large-scale, multicenter, randomized controlled trials that include diverse patient populations and clinical settings. Such studies could provide stronger evidence for integrating minimal stimulation protocols into routine clinical practice. Longitudinal studies examining cumulative live birth rates from all frozen embryos, as well as child health outcomes, would offer a more holistic view of treatment success. Additionally, cost-effectiveness analyses comparing minimal and high-dose stimulation could inform policy decisions and improve access to care in resource-constrained environments. Incorporating patient-reported outcomes such as emotional well-being, treatment satisfaction, and overall experience would further strengthen the evidence for patient-centered care. Finally, mechanistic studies exploring how stimulation intensity influences oocyte and embryo competence at the molecular level could provide valuable insights into optimizing individualized stimulation protocols.

## 5. Conclusions

In conclusion, this study contributes to the evolving paradigm of ovarian stimulation in women of advanced maternal age undergoing preimplantation genetic screening. By demonstrating that minimal stimulation protocols can achieve reproductive outcomes on par with high-dose regimens, the study underscores the importance of prioritizing embryo competence over oocyte quantity. These findings support the growing recognition of minimal stimulation as a viable, patient-friendly, and cost-conscious alternative to conventional strategies. As infertility management continues to evolve, such protocols may offer a more personalized and physiologically balanced approach, particularly for older patients seeking effective and less burdensome treatment options.

## Figures and Tables

**Table 1:** Intergroup comparison of baseline characteristics, laboratory parameters, and clinical outcomes between minimal and high stimulation protocols in advanced maternal age women undergoing preimplantation genetic screening.

	Groups		Test Statistics	
	MSP *n* = 99	HSP *n* = 99	Test Value	*p* Value
**Age**	40.4 ± 1.5	40.2 ± 1.5	0.875	0.383 †
**BMI**	25.96 ± 4.93	26.45 ± 4.27	0.738	0.461 †
**AFC**	3.0 (2.0)	4.0 (2.0)	2.486	**0.013** ^&^
**M2 (Mature)**	3.0 (2.0)	4.0 (1.0)	2.998	**0.003** ^&^
**ENDO**	10.66 ± 1.60	11.11 ± 1.92	1.810	0.072 †
**FSH**	9.26 ± 2.14	8.74 ± 2.23	1.694	0.092 †
**LH**	7.73 ± 1.55	8.08 ± 1.42	1.673	0.096 †
**E2**	40.00 (32.00)	33.00 (17.00)	1.830	0.067 ^&^
**AMH**	0.50 (0.60)	0.70 (0.60)	1.085	0.278 ^&^
**LBR per EE**	45.7	45.6	0.895	0.22
**PGD**, n (%)				
Normal	35 (35.4)	37 (37.4)	0.807	0.768 ‡
Broken	64 (64.6)	62 (62.6)		
**Beta**, n (%)				
There is pregnancy	23 (67.6)	25 (69.4)	0.001	>0.999 Փ
No pregnancy	11 (32.4)	11 (30.6)		
**Sac**, n (%)				
There is	20 (58.8)	21 (58.3)	0.001	>0.999 Փ
None	14 (41.2)	15 (41.7)		
**Birth**, n (%)				
There is	16 (47.1)	18 (50.0)	0.001	0.995 Փ
None	18 (52.9)	18 (50.0)		

*n*: Number of patients; %: column percentage, numerical variables are given as mean ± standard deviation or median (interquartile range) value. †: *T*-test for independent samples; ^&^: Mann–Whitney U test; ^‡^: Pearson chi-square test, Փ: Yates chi-square test. LBR: live birth rate, EE: Euploid Embryo.

## Data Availability

The data generated in the present study may be requested from the corresponding author.

## Abbreviations

The following abbreviations are used in this manuscript:

AMA	Advanced Maternal Age
ART	Assisted Reproductive Technologies
AFC	Antral Follicle Count
AMH	Anti-Müllerian Hormone
BMI	Body Mass Index
CC	Clomiphene Citrate
CDC	Centers for Disease Control and Prevention
EE	Euploid Embryo
E2	Estradiol
ET	Embryo Transfer
FET	Frozen Embryo Transfer
FSH	Follicle-Stimulating Hormone
GnRH	Gonadotropin-Releasing Hormone
hCG	Human Chorionic Gonadotropin
hMG	Human Menopausal Gonadotropin
ICSI	Intracytoplasmic Sperm Injection
IVF	In Vitro Fertilization
LBR	Live Birth Rate
LH	Luteinizing Hormone
MSP	Minimal Stimulation Protocol
M2	Mature Oocyte
NGS	Next-Generation Sequencing
OHSS	Ovarian Hyperstimulation Syndrome
PGD	Preimplantation Genetic Diagnosis
PGS	Preimplantation Genetic Screening
